# Patient-reported quality of life after primary major joint arthroplasty: a prospective comparison of hip and knee arthroplasty

**DOI:** 10.1186/s12891-015-0814-9

**Published:** 2015-11-26

**Authors:** Zoe H. Dailiana, Ippolyti Papakostidou, Sokratis Varitimidis, Lycurgos Liaropoulos, Elias Zintzaras, Theofilos Karachalios, Emmanuel Michelinakis, Konstantinos N. Malizos

**Affiliations:** Department of Orthopaedic Surgery, Faculty of Medicine, University of Thessalia, 3 Panepistimiou Street Βiopolis, 41500 Larissa, Greece; Center for Health Services Management and Evaluation, Faculty of Nursing, University of Athens, Athens, Greece; Department of Biomathematics, Faculty of Medicine, School of Health Sciences, University of Thessalia, 3 Panepistimiou Street, Βiopolis, 41500 Larissa, Greece; Department of Orthopedics, NIMITS Hospital, 10 Monis Petraki Street, Athens, Greece

**Keywords:** Hip osteoarthritis, Knee osteoarthritis, Total Hip Arthroplasty, Total Knee Arthroplasty, Quality of Life

## Abstract

**Background:**

To investigate and compare the impact of primary hip (THA) and knee (TKA) arthroplasty on quality of life in patients with osteoarthritis, to determine patients’ satisfaction with total joint arthroplasty, and to detect the effect of patients’ demographic and clinical characteristics on outcome.

**Methods:**

Three hundred seventy eight (378) patients with hip (174) and knee (204) osteoarthritis undergoing total joint arthroplasty (174 THA-204 TKA) were assessed pre- and post-operatively (6 weeks, 3, 6, and 12 months) using the Western Ontario and McMaster Osteoarthritis Index (WOMAC) and Centre for Epidemiological Studies Depression Scale (CES-D10). The patients’ satisfaction with the results of total joint arthroplasty was also assessed. Differences were analyzed using general linear model for repeated measures.

**Results:**

The one-year response rate was 97 % for THA and 90 % for TKA. WOMAC and CES-D10 scores improved significantly after one year for both THA and TKA (*P* < 0.0001). The improvement in WOMAC total score was significantly greater for TKA patients (*P* < 0.0001 at 12 months). WOMAC pain and stiffness improved earlier for THA (6 weeks), while TKA had equivalent improvements at 3 and 6 months respectively. Both THA/TKA displayed significant improvement of WOMAC function at 3 months but TKA had greater improvement. Age, body mass index, residence, education and social support were not significant predictors of quality of life after total joint arthroplasty. One year postoperatively 88 % of patients were satisfied.

**Conclusions:**

WOMAC and CES-D10 improved significantly one year postoperatively. Although pain and stiffness improved earlier in THA, functional improvement was inferior in THA compared to TKA.

## Background

Osteoarthritis (OA) is one of the most common diseases affecting the musculoskeletal system in elderly people and has substantial impact on patients’ quality of life (QoL) [1].

Total hip arthroplasty (THA) and total knee arthroplasty (TKA) provide long-lasting joints that relieve pain and improve physical function [2–7]. While some authors report similar or equivalent outcomes for THA and TKA [8, 9], other authors report slower recovery after TKA [6, 10–12]. Most clinical studies also investigated discrepancies in outcomes after total joint arthroplasty (TJA) and have concluded that demographic factors such as gender, age, body mass index (BMI), socioeconomic status and depression can impact patient-perceived outcomes [13, 14].

This study analyses prospectively the QoL after TJA. The objectives were: (1) to investigate and compare the impact of THA and TKA on three dimensions of QoL in patients with OA, (2) to detect the effect of patients’ demographic and clinical characteristics on QoL outcomes 12 months after TJA and (3) to determine patients’ satisfaction with TJA.

## Methods

Patients admitted sequentially to two Greek hospitals (University Hospital of Larissa and Veteran’s Hospital in Athens) for unilateral, primary, elective THA or TKA were asked to participate to the study and to provide written consent. Exclusion criteria were arthritis due to inflammatory diseases, developmental dysplasia of the hip, severe cardiac, neurological and psychiatric comorbidities and inability to communicate in Greek. Of 412 eligible patients, 8 refused to participate, and 26 were excluded because of contralateral TJA during follow-up. The final study cohort consisted of 378 patients (174 THA and 204 TKA). Patients’ characteristics are summarized in Table 1. All arthroplasties were performed by the same groups of surgeons in the two hospitals, with consistent technique (THA: posterior approach, uncemented technique; TKA: anterior, medial parapatellar approach, cruciate retaining, cemented technique, no patella resurfacing). After discharge from the hospital the patients received only mild analgesics (paracetamol/acetaminophen) occasionally. The majority of the TKA patients (176) followed a rehabilitation program at home whereas most THA patients (155) did not use rehabilitation services. Twenty-eight of 204 patients (13.7 %) with TKA and 19 of 174 patients (10.9 %) with THA were transferred to rehabilitation centers.

**Table 1:** Characteristics of participants

	TKA (*n* = 204)	THA (*n* = 174)
Demographics		
Age, mean ± SD years	69.17 ± 6.69	64.98 ± 11.1
Female, No. (%)	162 (79.4)	118 (67.8)
Absence of social support, No. (%)	36 (17.6)	28 (16)
Education level		
Elementary or less, No. (%)	130 (63.7)	97 (55.7)
Residence: Rural, No. (%)	86 (42.1)	37 (21.3)
Medical Status		
BMI, mean ± SD kg/m^2^		
≥ 30, No. (%)	108 (52.9)	48 (23.4)
Charlson comorbidity scale*	1.6 (1.5)	1.2 (0.7)
LOS (days)	6.68 ± 1.3	8.0 ± 3.1

*BMI* body mass index, *LOS* length of stay

*Charlson comorbidity scale, 0–27 (higher scores indicate more comorbid illness)

### Study design and data collection

The study design was prospective, with baseline (day before surgery) and follow-up contacts at 6 weeks, 3, 6, and 12 months postoperatively. At baseline, a structured questionnaire recorded information on age, gender, educational level, place of residence, and social support.

Residence was determined as urban/semi-urban and rural on the basis of the 2001 census. The patient population of the University Hospital originated from rural and urban/semi-urban areas while the population of the Veteran’s Hospital originated from urban areas.

The patients’ social support was determined by their marital and living status [15], and their educational level was coded as either low (primary school-less) or high (secondary school-higher). The high illiteracy rates in the elderly (13.6 %) forced us to design the data collection accordingly.

The clinical parameters included specific diagnosis, BMI and Charlson Comorbidity Index score [16]. Length of hospital stay was also recorded. Information regarding readmissions, post-hospital care and rehabilitation were gathered at the follow-up interviews.

The questionnaires were administered in face-to-face interviews by one investigator (IP) who was not involved in the direct care of the patients. The study was approved from the scientific committee of the University hospital and the patients provided written consent to participate to the study and to the follow-up evaluations.

### QoL measurements

Pain and functional impairment were assessed by the WOMAC Osteoarthritis Index [17], and depression by the CES-D10 (a validated cut off score of 10 differentiates clinically depressed from non-depressed patients) [18]. Patient satisfaction with the results of TJA was assessed in three aspects: overall satisfaction, satisfaction with pain relief, and satisfaction with functional improvement/ability to perform daily activities. Patients were categorized as very/mostly satisfied, somewhat satisfied, and dissatisfied [19, 20].

### Data analysis

Means and proportions were calculated separately for THA/TKA patients; then, the two groups were compared in terms of their preoperative characteristics using *t*-test and chi-square test, respectively. The following variables were defined: age (under/over 65), gender, BMI (under/over 30 kg/m^2^), level of education (low/high), place of residence (rural/urban-semi), social support (married-living with someone/otherwise), preoperative WOMAC and CES-D10 scores.

The pre- and post-operative effects in WOMAC and CES-D10 scores were compared using a general linear model for repeated measures. Pair-wise comparisons were tested using post hoc t-tests with Bonferonni’s correction.

Effect size (ES), a measure of the relative magnitude of a change, was calculated as the difference between the preoperative and 12-month postoperative scores divided by the SD of the preoperative scores. Effect sizes of 0.2, 0.5 and 0.8 indicate small, medium and large degrees of change, respectively. An analysis of variance was used to estimate the effects of all factors of interest on WOMAC dimensions at 12 months postoperatively. Statistical analysis was performed with the use of SPSS (version 15.0; SPSS, Chicago, IL, USA), and statistical significance was set at 5 % (*P* <0.05).

### Results

The response rates at 6 weeks, 3 months, 6 months and 12 months follow-up were 100 %, 99.5 %, 99 % and 97.1 % for THA patients and 98.5 %, 97 %, 94.6 %, and 90.2 % for TKA patients respectively.

### Patients’ characteristics

TKA patients were significantly older (*P* < 0.0001) and overweight (BMI ≥ 30 kg/m^2^; *P* < 0.0001) at operation compared to THA patients (Table 1). The majority of them lived in rural areas (*P* < 0.0001) and displayed a lower education profile with 16 % declaring illiterate. The corresponding percentage of illiteracy in the THA patients was 7 % (*P* < 0.0001) (Table 1). TKA patients also experience a significantly shorter hospital stay (*P* = 0.001).

### Preoperative QoL

**Fig. 1:**
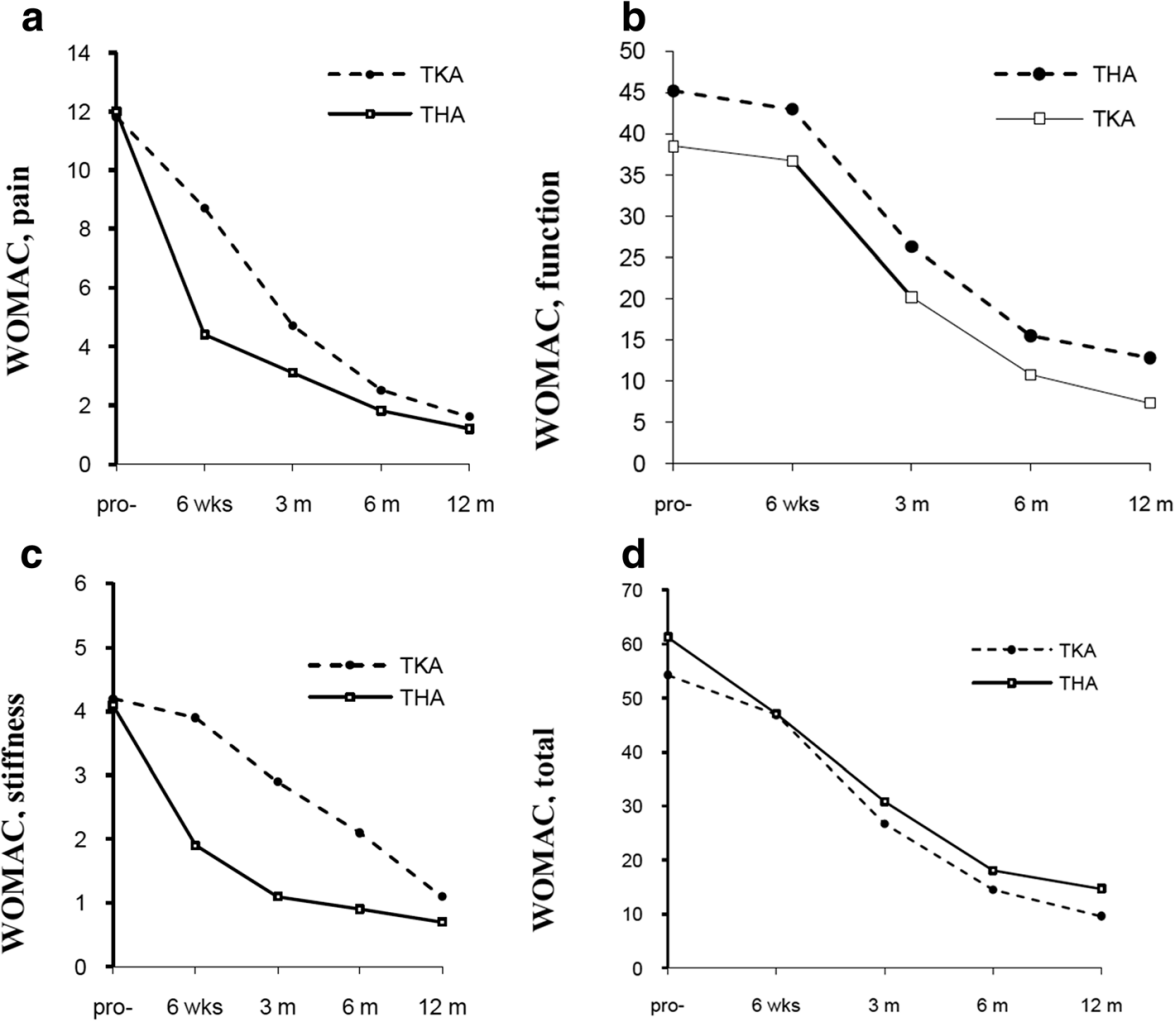
(**a**, **b**, **c**, **d**) Pre-and postoperative scores of WOMAC pain, function and stiffness domains and total score in patients undergoing TKA/THA

Preoperative WOMAC total score for hip OA patients was significantly higher (worse) compared to knee OA patients (*P* < 0.0001; Table 2, Fig 1d). Significantly higher function scores were recorded in the hip OA patients compared to knee OA patients (Fig 1b), although the pain and stiffness dimensions did not differ significantly between the two groups (Fig.1a, c).

**Table 2:** Prospectively tabulated WOMAC hip and knee total scores

WOMAC total score	*N*	THA	*N*	TKA	*P*
Preoperatively	174	61.3 ± 15.8	204	54.3 ± 14.4	<0.0001
6-wks post-op	174	47.1 ± 12.0	01	46.9 ± 15.9	0.9
3-m post-op	173	30.9 ± 10.2	198	26.7 ± 12.6	0.001
6 m post-op	172	18.1 ± 12.2	193	14.5 ± 12.1	0.007
12 m post-op	169	14.7 ± 9.7	184	9.6 ± 10.0	<0.0001
WOMAC total change score					
Pre-op – 12 months^a^		46.6(43.4–49.8)		44.7(41.3–48.0)	
ES Pre-op – 12 months		2.9		3.1	

WOMAC total score: 0–96, higher scores indicate greater difficulty

*ES* = effect size

^a^Values are mean (95 % confidence interval)

The analysis of WOMAC preoperative dimensions showed that for patients undergoing TJA, women displayed significantly worse pain (*P* = 0.01 and *P* = 0.007 respectively) and function scores (*P* = 0.002 and *P* = 0.001 respectively) compared to men. Obese patients had worse pain (*P* = 0.004 and and *P* = 0.04 respectively) and poorer function scores (*P* < 0.0001 and *P* = 0.01 respectively) than non-obese (Table 3).

**Table 3:** WOMAC dimension scores at baseline and 12 months after THA and TKA

	THA WOMAC^a^						TKA WOMAC^a^					
	PAIN		FUNCTION		STIFFNESS		PAIN		FUNCTION		STIFFNESS	
	Pre-op	12 m	Pre-op	12 m	Pre-op	12 m	Pre-op	12 m	Pre-op	12 m	Pre-op	12 m
		post-op		post-op		post-op		post-op		post-op		post-op
GENDER												
Men	10.7 (3.9)	1.0 (2.1)	42.3 (4.8)	12.6 (9.3)	3.0 (1.1)	0.9 (1.1)	10.3 (4.5)	1.6 (2.4)	34.7 (14.1)	7.5 (9.4)	3.6 (1.8)	0.7 (0.9)
Women	12.5 (3.5)*	1.3 (2.2)	46.1 (5.7)**	12.8 (8.0)	4.7 (1.5)	0.7 (1.0)	12.2 (3.8)*	1.4 (2.4)	39.6 (10.1)*	7.2 (8.1)	4.2 (1.5)	0.8 (1.1)
AGE												
< 65	12.0 (3.6)	1.4 (1.5)	45.1 (5.7)	12.4 (8.1)	4.0 (1.4)	0.8 (0.9)	12.4 (3.4)	1.3 (1.9)	37.8 (12.4)	5.3 (6.7)	3.9 (1.7)	0.7 (0.9)
≥ 65	11.9 (3.7)	1.0 (1.1)	45.2 (5.5)	13.1 (8.6)	4.1 (1.5)	0.7 (1.0)	11.6 (4.2)	1.7 (2.5)	38.6 (10.9)	7.9 (8.8)	4.1 (1.6)	0.9 (1.1)
BMI												
< 30	10.6 (3.8)	1.0 (1.0)	44.0 (5.3)	12.2 (8.6)	4.2 (1.5)	0.7 (0.6)	11.1 (4.1)	1.5 (2.2)	36.2 (11.6)	6.4 (8.1)	3.9 (1.6)	0.8 (1.0)
≥ 30	13.2 (3.1)**	1.7 (1.8)	48.3 (5.4)**	14.3 (9.0)	4.1 (1.4)	0.9 (0.8)	12.4 (3.8)*	1.7 (2.6)	40.5 (10.6)*	7.9 (8.6)	4.2(1.6)	0.9 (1.0)
EDUCATION												
Low (elementary or less)	13.1 (4.1)	0.7 (0.1)	46.3 (5.3)	10.9 (8.8)	4.1 (1.6)	0.4 (0.3)	12.3 (4.2)	1.1 (1.5)	42.1 (10.7)	6.4 (7.7)	4.6 (1.3)	0.9 (1.1)
High	11.9 (3.6)	1.2 (0.1)	45.1 (5.6)	11.8 (8.6)	4.0 (1.3)	0.8 (0.6)	11.7 (3.9)	1.7 (2.5)	37.9 (11.3)	7.4 (8.5)	3.9 (1.6)	0.5 (0.7)
SOCIAL SUPPORT												
Yes	11.8 (3.7)	1.3 (1.1)	45.1 (5.8)	13.1 (7.0)	4.0 (1.4)	0.8 (0.7)	11.8 (4.1)	1.5 (2.3)	38.3 (11.9)	7.0 (8.5)	4.1 (1.6)	0.8 (1.0)
No	12.8 (3.6)	0.9 (1.4)	45.6 (4.8)	11.2 (6.9)	4.3 (1.5)	0.5 (0.3)	11.7 (3.5)	2.1 (2.9)	39.5 (7.3)	8.4 (8.1)	3.9 (1.6)	0.9 (1.1)
RESIDENCE												
Rural	12.9 (3.7)	1.4 (1.5)	45.4 (5.9)	14.0 (9.8)	4.1 (1.2)	0.9 (1.0)	11.7 (4.5)	1.7 (2.4)	38.7 (12.7)	7.2 (8.1)	4.1 (1.5)	0.9 (1.1)
Urban/semi-	11.7 (3.6)	1.1 (1.1)	45.1 (5.5)	12.3 (8.4)	4.1 (1.0)	0.7 (1.0)	11.9 (3.7)	2.1 (2.8)	38.4 (10.3)	8.3 (9.3)	4.0 (1.7)	0.7 (0.9)

WOMAC scores: pain 0–20, function 0–68, stiffness 0–8; higher scores indicate greater difficulties

^a^Values are mean ± SD, *P ≤ 0.05, **P ≤ 0.005

**Fig. 2:**
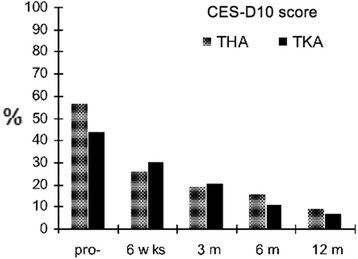
Patients with depression according to the pre- and post-operative CES-D10 scores

Depression according to CES-D10 (score > 10) was detected in 56.6 % of hip OA patients and in 44.4 % of knee OA patients (Fig 2).

### Postoperative changes in QoL over time

Repeated measures ANOVA showed that the WOMAC total score changed significantly (*P* < 0.0001) from preoperative to 6 weeks, 3, 6 and 12-month postoperative evaluations for both groups (THA, TKA). The ES at 12 months for the WOMAC total score were 2.9 in THA and 3.1 in TKA, and showed an improvement of 76 % and 82.3 % respectively (Table 2). Comparisons between THA and TKA groups at each time point are shown in Table 2 and Fig 1d. The improvement in WOMAC total score was significantly greater for TKA patients compared to THA patients in all time points (3, 6, 12 months) except the 6 weeks evaluation (Table 2).

There was no significant change in WOMAC function scores between preoperative and 6 weeks postoperative assessment in both patient groups. After 6 weeks both THA and TKA patients reported significant improvement, but function scores were significantly better in TKA group (3, 6, 12 months) (Fig 1b).

According to WOMAC pain and stiffness dimensions THA patients displayed significantly lower scores (improvement) compared to TKA patients at 6 weeks, 3, and 6 months postoperatively. Pain and stiffness improved earlier for THA (6 weeks). TKA had equivalent improvements at 3 months for pain and at 6 months for stiffness. At 12 months postoperatively, pain and stiffness scores were similar (Fig 1a, c).

The ES and % improvement for THA and TKA were 2.6 (73.9 %) and 2.8 (81 %) respectively for function, 2.9 (90 %) and 2.5 (86.3 %) for pain, and 2.2 (81 %) and 2.0 (78.6 %) for stiffness.

The analysis of the WOMAC postoperative dimensions according to demographic factors showed no significant differences in THA patients in all postoperative intervals. A significant difference was observed in the TKA group between men and women at the first postoperative review (6 weeks). WOMAC pain (*t* = 2.71; *P* = 0.007) and function scores (*t* = 3.71; *P* < 0.001) were significantly better in men. Regarding the three WOMAC dimensions, the multivariable analysis showed that the only predictive variables of better outcome were the baseline scores for WOMAC pain (*P* = 0.03), function (*P* = 0.01) and stiffness (*P* = 0.04) in the THA group and the baseline scores for WOMAC pain (*P* = 0.02) and function (*P* = 0.002) in the TKA group (Tables 4, 5).

**Table 4:** Multivariable analysis of post-operative (12 months) changes in THA WOMAC domains

	WOMAC^a^								
	Pain			Function			Stiffness		
Variables	Diff^b^	95 % CI	*P*-value	Diff	95 % CI	*P*-value	Diff	95 % CI	*P*-value
Gender									
Male vs Female	0.09	−0.58, 0.60	0.9	−2.12	−5.00, 0.8	0.2	−0.48	−1.19,1.16	0.2
Age									
< 65 vs ≥65 vs	−0.21	−1.90, 0.47	0.5	2.33	−1.06, 5.73	0.2	−0.14	−0.19, 0.47	0.4
BMI									
< 30 vs ≥30	−0.29	−0.99, 0.40	0.4	−1.73	−5.29, 1.81	0.3	−0.19	−0.53, 0.14	0.3
Education									
Low^c^ vs High	−0.35	−1.55, 0.84	0.6	0.85	−5.07, 6.78	0.7	0.09	−0.67, 0.49	0.7
Social Support									
Yes vs No	−0.38	−1.13, 0.35	0.3	−0.19	−3.88, 3.51	0.9	−0.15	−0.51, 0.20	0.4
Residence									
Rural vs Urban/semi	−0.43	−0.38, 1.24	0.3	0.20	−3.83, 4.23	0.9	−0.22	−0.17, 0.61	0.3
Pre-intervention Pain	0.11	2.29, 0.02	0.03	-	-	-	-	-	-
Pre-intervention Function	-	-	-	0.21	0.05, 0.36	0.01	-	-	-
Pre-intervention Stiffness	-	-	-	-	-	-	0.94	−0.02, 0.18	0.04

^a^WOMAC scores: pain 0–20, function 0–68, stiffness 0–8; higher scores indicate greater difficulty

^b^Estimated differences between categories after adjustment by all other variables

^c^Elementary or less; − Not applicable

**Table 5:** Multivariable analysis of post-operative (12 months) changes in TKA WOMAC domains

	WOMAC^a^								
	Pain			Function			Stiffness		
Variables	Diff^b^	95 % CI	*P*-value	Diff	95 % CI	*P*-value	Diff	95 % CI	*P*-value
Gender									
Male vs Female	0.02	0.84, 0.88	0.2	1.30	−1.68, 4.28	0.4	−0.15	−0.52, 0.23	0.4
Age									
< 65 vs ≥65	−0.48	1.28, 0.31	0.9	−2.47	−5.23, 0.28	0.07	−0.18	−0.53, 0.17	0.3
BMI									
< 30 vs ≥30	−0.15	0.85, 0.54	0.6	−0.97	−3.41, 1.46	0.4	−0.06	−0.37, 0.24	0.4
Education									
Low^c^ vs High	−0.72	1.69, 0.44	0.06	−3.08	−6.76, 0.59	0.09	−0.16	−0.70, 0.28	0.8
Social Support									
Yes vs No	−0.86	1.82, 0.09	0.07	−2.24	−5.55, 1.06	0.1	−0.17	−0.59, 0.25	0.4
Residence									
Rural vs Urban/semi	−0.52	1.10, 0.42	0.05	−2.25	−4.87, 0.38	0.09	−0.15	−0.59, 0.22	0.06
Pre-intervention Pain	0.10	2.29, 0.02	0.02	-	-	-	-	-	-
Pre-intervention Function	-	-	-	0.17	0.06, 0.28	0.002	-	-	-
Pre-intervention Stiffness	-	-	-	-	-	-	0.07	−0.02, 0.17	0.1

^a^WOMAC scores: pain 0–20, function 0–68, stiffness 0–8; higher scores indicate greater difficulty

^b^Estimated differences between categories after adjustment by all other variables

^c^Elementary or less; − Not applicable

According to CES-D10 scores, both THA and TKA patients showed a significant reduction of depression 12 months postoperatively. TKA patients showed a significant reduction between the 5 time points tested (*P* < 0.001). In the THA group a significant improvement was observed between preoperative and 6 weeks postoperative scores (*P* < 0.001) and between 3 and 6 months postoperative scores (*P* = 0.03). Between group comparisons showed that THA patients displayed significantly better CES-D10 scores (mean 5.1) compared to TKA patients (mean 7.6; *P* < 0.001) 6 weeks postoperatively. After 6 weeks there was no significant difference between THA and TKA groups. Twelve months postoperatively 9.8 % of THA patients and 7.4 % of TKA patients remain depressed (Fig 2).

Patients’ satisfaction is shown in Table 6. Overall satisfaction 12 months postoperatively revealed 87.5 % of very/mostly satisfied THA patients and 2.4 % dissatisfied. The corresponding rates for TKA patients were 88 % and 5.5 %. Each group was satisfied with a different parameter: THA patients from pain relief and TKA patients from functional improvement.

**Table 6:** Level of patient satisfaction following TKA/THA at 3, 6, and 12 months postoperatively

	TKA								
Level of Satisfaction (%)	Over all			Pain relief			Function restore		
	3 m	6 m	12 m	3 m	6 m	12 m	3 m	6 m	12 m
1. Very satisfied/mostly satisfied	52.7	82.4	88	64.1	80.8	83.7	70.7	89	89.1
2. Somewhat satisfied	31.9	9.9	6.5	30.8	14	11.4	27.3	8.9	7.6
3. Dissatisfied	15.4	7.7	5.5	5.1	5.2	4.9	2.0	2.1	3.3
	THA								
Level of Satisfaction (%)	Over all			Pain relief			Function restore		
	3 m	6 m	12 m	3 m	6 m	12 m	3 m	6 m	12 m
1. Very satisfied/mostly satisfied	78.9	91.6	87.5	77.8	81.7	96	60.3	87.7	86.7
2. Somewhat satisfied	20.1	6.9	10.1	17.8	14.3	3.5	38.2	10.3	9.8
3. Dissatisfied	1.0	1.5	2.4	4.4	4.0	0.5	1.5	2.0	3.4

## Discussion

The present study evaluated and compared prospectively the QoL after TKA and THA, as well as the effect of socio-demographic characteristics on the outcome.

Our finds are consistent with those in studies demonstrating large treatment effects in terms of QoL after THA or TKA [4–7, 21]. Nonetheless there is controversy whether THA or TKA provides greater or similar improvement. Several studies have reported slower recovery and inferior outcomes in pain and function for TKA compared with THA [6, 10–12], while other studies report similar outcomes for TKA and TKA [8, 9].

Our study demonstrated that relief from pain and stiffness were not only greater after THA but also occurred more rapidly. As early as 6 weeks postoperatively, THA patients show significant improvements in pain and stiffness, whereas TKA patients did not demonstrate equivalent improvements and obtained the respective levels of pain and stiffness reduction at 3 and 6 months. The gains in physical function after TKA and THA are delayed compared to pain relief; these are significant the 3^rd^ postoperative month for both groups.

In contrast to previous reports, TKA patients displayed greater improvement in function when compared to THA at 3, 6, 12 months. A possible explanation could be the higher rates of functional difficulties reported by THA patients preoperatively and thus, despite the significant postoperative improvement they could not reach the improvement of TKA patients.

Although depressive mood was detected in more than 44 % of the TKA patients and 56 % of the THA patients preoperatively, the CES-D10 score improved similarly in the hip and knee patients12 months postoperatively.

The influence of gender, age and BMI on the patient-perceived outcomes after TJA has been reviewed in the literature [13, 14, 22–25]. Our study, in accordance with other studies demonstrated that women had worse pain and disability than men at the time of TJA [21, 26, 27]. Postoperatively, these gender differences were present only after TKA at the first follow-up (6 weeks) [21]. After that period women had greater improvement than men [26, 28]. The preoperative gender differences could be attributed to a delayed access of women to surgical management because of greater fear or to avoid the burden on the family after surgery [11, 12].

Contrary to other studies showing that older age negatively influences the clinical outcome we found that patients over and under the 65 years experience similar benefits and recovery [13, 14]. However, the small number of patients over 80 years in our study precludes any definite conclusions, whereas the absence of significant comorbidities in the patients of our series possibly indicates a selection bias [26].

Studies evaluating the effect of BMI on the outcome of TJA have been inconclusive and contradictory [22–25, 29, 30]. In this study, obese patients reported more pain, functional limitations, and depressed mood preoperatively, but no difference was observed between these patients, according to WOMAC and CES-D scores 12 months postoperatively, in both procedures, suggesting that obesity is not related to the short-term outcome [21, 29, 30]. However, morbidly obese patients (BMI > 40 kg/m^2^) made up only a small subset of the sample (1.5 %).

Rural–urban disparities in access to and utilization of medical care have been focus of concern [31]. We hypothesized that rural residents, which tend to be in a lower socioeconomic status, may have underutilization of health care services and these disparities might affect the TJA outcome. We hypothesized also that lower education could affect the compliance with medical instructions or the timely seeking of medical care. Our findings did not support our hypothesis. No significant disparities were present between rural and urban residences, and low education was not found to be a predictor of poor outcome. A possible explanation is that access to the public medical services is not limited for patients of lower socioeconomic background. In addition, routine visits to the outpatient department during the follow-up period offers a close patient-surgeon contact.

Social support is another important factor that influences the QoL after TJA, through assistance and support during the recovery period [32]. In contrast to other studies we found that patients not married or living alone did not have worse QoL compared to married/living with others patients. We believe that this finding is most likely related to the help from social environment (family members, friends, neighbours), which is strong in our country and compensates the absence of formal public community services.

The strengths of the study are its prospective design, the high rates of return to follow-up and the use of a trained independent research assistant that recruited patients and followed them at each assessment. However we acknowledge that this study presents certain limitations such as the involvement of only two centres; therefore a multicenter research is needed for generalization of the results. Finally, the low proportion of males and the narrow age range of our patients, limited the usefulness of the results with respect to gender and age. The measure of social support in this study was crude, simply using patients-reported preoperative living and marital status. Further studies need to explore these variables.

## Conclusion

In all patients WOMAC and CES-D10 scores improved significantly one year postoperatively. Patients with THA had earlier pain relief and stiffness improvement than TKA patients, who on the contrary achieved better functional improvement.

### Availability of data and materials

All data sets on which the conclusions of the paper rely may be found at the site of the Greek National Archive of PhD theses (this study is part of the dissertation of Dr Papakostidou) at the following address: www.didaktorika.gr/eadd/handle/10442/28083.
